# Isolation, Characterization, and Identification Candidate of Probiotic Bacteria Isolated from Wadi Papuyu (*Anabas testudineus* Bloch.) a Fermented Fish Product from Central Kalimantan, Indonesia

**DOI:** 10.1155/2022/4241531

**Published:** 2022-05-05

**Authors:** Yulistia Budianti Soemarie, Tiana Milanda, Melisa Intan Barliana

**Affiliations:** ^1^Department of Biological Pharmacy, Faculty of Pharmacy, Universitas Padjadjaran, Raya Jatinangor Street, Hegarmanah, Jatinangor, Sumedang, West Java, Indonesia; ^2^Department of Biological Pharmacy, Faculty of Pharmacy, Universitas Islam Kalimantan Muhammad Arsyad Al Banjari, Adhyaksa Kayutangi, Sungai Miai, North Banjarmasin, Banjarmasin City, South Borneo, Indonesia; ^3^Centre of Excellence in Higher Education for Pharmaceutical Care Innovation, Universitas Padjadjaran, Bandung, Indonesia

## Abstract

During the wadi fermentation process, some microorganisms can grow, including lactic acid bacteria (LAB), affecting the taste and texture of the final product. Some LAB strains are used as probiotics such as the *Lactobacillus* and *Bifidobacterium* groups. This study aimed at isolating, in vitro characterizing, and identifying microbial isolates from wadi papuyu (*Anabas testudineus* Bloch.). The stages started from sample collection, manufacture of wadi papuyu by fermentation for 8 days, isolation of bacteria from wadi papuyu, in vitro characterization, and identification of bacterial isolates with VITEK 2 Compact and PCR-sequencing methods 16S rRNA and 18S rRNA. The number of microbial colonies growing on MRS agar and MHA was 22 in total, while after purification and characterization it was observed only 4 different microbial isolates. Candidates are tested to determine whether they meet the criteria to be candidates for probiotic cultures. The in vitro testing of four isolates showed that they do not possess probiotic characteristics, especially in *autoaggregation* tests. Identification results using the VITEK 2 Compact method and 16S rRNA gene PCR-sequencing showed that of the 4 isolated strains, three were bacterial and one belonged to *yeasts*.

## 1. Introduction

Fermentation has been one of the most widely used traditional preservation methods since ancestral times. Several countries including Indonesia produce a lot of fermented foods, especially fish. The fermentation method often involves salting and acidification. Fish fermentation is defined as the process of preserving fresh fish that undergoes a series of biochemical changes caused by microorganisms or enzymes. These changes include acidification, gelation of myofibrillar and sarcoplasmic muscle proteins, and protein and fat degradation. In some cases, the microbiota can also produce antimicrobial substances that can help improve safety and nutritional value and extend the shelf life of food [1, 2].

Wadi is a fermented fish product that is in great demand by the people of South and Central Kalimantan, Indonesia [3, 4]. It is produced using one of the traditional fermentation processes, and it is nearly black (close to the color of fresh fish), with a clay texture, a distinctive fermented aroma, and a salty taste. This salty taste is caused by the addition of salt, sugar, and *samu/lumu* during the wadi processing and storage at room temperature for 7 × 24 hours [5]. In addition to salt, another additive used in the fermentation of this fish wadi is *roasted-mashed rice* called s*amu/lumu*. Wadi is usually made from freshwater fish such as papuyu (*Anabas testudineus* Bloch.) [6]), which is commonly found in Central Kalimantan and lives in swamps or other stagnant water [7]. Papuyu is favored by people because this fish meets the needs of animal protein intake at affordable prices and has a delicious taste. Wadi papuyu has a distinctive taste and smell of fermentation which is very much liked by the people of Kalimantan. Wadi is usually processed by frying, grilling, or cooking with other additives. In this study, wadi papuyu were used that had not undergone any processing; this was to keep the microbes contained in wadi papuyu alive and not damaged by the processing.

Additionally, food fermentation products (such as wadi) use lactic acid bacteria (LAB) during fermentation which change the product characteristics by producing lactic acid, specific enzymes, and aromatic compounds [8]. In a previous study, isolation and identification of bacteria in Betok fish (a genus of *Anabas*) were dominated by *Acinetobacter*, *Enterobacteriaceae*, and *Brucella*; these three bacteria are pathogenic [9]. LAB obtained from other fermented fish products is Rusip from Lampung, Indonesia. LAB strains produced by Rusip are *Streptococcus* sp., *Lactobacillus* sp., and *Leuconostoc* sp. [10]. Traditional fermented foods can be used as a potential source of LAB which can be applied as probiotics.

Furthermore, probiotics are defined as food supplements of live microorganisms, administrated in sufficient quantities with positive influence on human health, and have the potential to be used as an alternative or supplement to antibiotics [11]. These microorganisms can change the food composition, replace the metabolic activity of the natural gut microbiota, and regulate the immune system [12]. LAB that have potential use as probiotics include *Lactobacillus* sp., *Pediococcus* sp., *Enterococcus* sp., *Weissella*, *and Leuconostoc* sp. [13–17]. LAB are usually isolated from fermented foods [18, 19] in certain environments such as high salinity and acidic conditions. Probiotic bacteria can act as immunostimulants and antibiotics and inhibit the growth of pathogenic bacteria [20].

Besides, consumer awareness of the importance of probiotics providing health benefits has resulted in large amounts of studies into LAB obtained from food fermentation. Therefore, this study is focused on identification of bacteria isolated from unprocessed wadi papuyu, as possible probiotic candidates.

## 2. Materials and Methods

### 2.1. General Experiment Procedures, Object Material, and Making of Wadi

**Figure 1:**
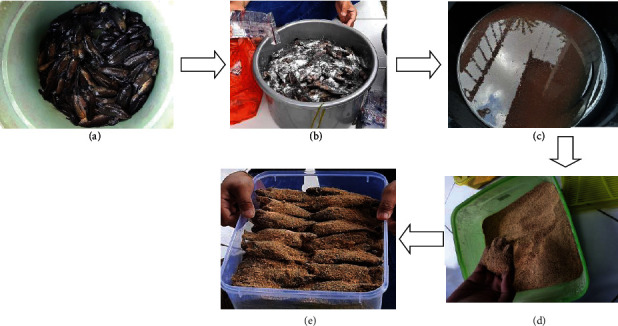
The process of making wadi papuyu (*Anabas testudineus* Bloch.): (a) fresh papuyu (*Anabas testudineus* Bloch.) fish used in making wadi; (b) the process of salting papuyu fish (left for 24 hours); (c) saltwater that comes out of the body of papuyu fish after being allowed to stand for 24 hours; (d) *samu/lumu* made from roasted-mashed rice; (e) Papuyu fish which was ready to be fermented into wadi for 7-14 days at room temperature.

Papuyu from the Fish Shelter at Palangkaraya City, Central Kalimantan, Indonesia, was use in this study. Wadi papuyu was made by first selecting the papuyu fish, then covering the fish with mashed rice and a pinch of salt, and arranging this neatly in a container. The container was taken to the laboratory and left at room temperature for 7 to 14 days with aerobic fermentation conditions at room temperature. Bacterial isolation was performed on the 8th day of wadi fermentation. The process of making wadi papuyu is shown in Figure 1.

### 2.2. Bacterial Isolation during Fermentation of Wadi

Bacteria were isolated from wadi papuyu using *de Man, Rogosa, and Sharpe* (MRS) and *Mueller Hinton* agar (MHA). To make a 10^−1^ to 10^−6^ dilution, one gram of wadi is diluted in 9ml of sterile distilled water. Subsequently, the plating was performed from a 10^−2^ dilution by taking 0.1 mL and spreading on sterile MRS and MHA petri dishes. Growing colonies on the selected media were then repurified by restreaking on MRS agar and MHA by quadrant streak method. Microbial isolates growing on MRS agar and MHA were then counted. The uniformity of the colony and its morphology was checked, after which the culture were stored in the MRS broth and Mueller Hinton agar (MHA) broth.

### 2.3. Characterization of Bacterial Isolates from Wadi

#### 2.3.1. Identification by Gram Staining, Observation of Bacterial Isolate Form, and Catalase Test

Characteristic testing was done by looking at the observation of bacterial form, gram staining, and catalase test. To see the bacterial form and gram staining is done by making a smear of each microbial isolate on a slide and viewed under a microscope. For the catalase test, a small amount of each microbial isolate was placed on a glass object slide, and one drop of 3% H_2_O_2_ reagent was added. Positive results are indicated by oxygen bubbles [21].

#### 2.3.2. Testing of Bacterial Isolates as Probiotics by In Vitro

*(1) Acid Tolerance*. Hundred *μ*L of each bacterial isolate was taken from its stock culture that had been rejuvenated in MRS broth at 37°C for 24 hours and transferred to 3 Eppendorf tubes containing 0.9 *μ*L MRS broth, which had been adjusted to pH 2, 3, and 4, respectively, and incubated at 37°C in a water bath for 3 hours. Then, the suspension was washed twice using sterile PBS and centrifuged at 3000 rpm for five minutes, and the supernatant was discarded. Fifty *μ*L of each pellet was inoculated in MRS broth and 5 *μ*L at neutral pH and incubated for 24-48 hours at 37°C anaerobic circumstances. Then, the growth of each LAB isolate was measured by a *spectrophotometer* (TECAN Infinite 200 PRO NanoQuant) at a wavelength of 660 nm (OD 660 nm) [22]. As for the isolates growing at pH 2 (according to the stomach pH), the number of microbes before and after incubation was determined after culturing on MRS agar [23].

#### 2.3.3. Aggregation Tests

*(1) Autoaggregation Test*. LAB isolates grown in MRS broth for 18 hours at a temperature of 37°C were centrifuged at 3500 rpm for 20 minutes, after which the precipitate was washed twice and resuspended with PBS until it reached 10^8^CFU/mL. *The autoaggregation test* was performed by measuring the initial and final absorbance (at 0 and 5 hours) after incubation at room temperature. Then 0.1mL of each LAB suspension was added into 3.9mL PBS and measured using *spectrophotometer* (TECAN Infinite 200 PRO NanoQuant) at a wavelength of 600nm. The percentage of *autoaggregation* was stated as follows [24]:

(1) $$\begin{array}{c} Autoaggregati\text{o}n\left(\%\right)=1-\left(\frac{A_{t}}{A_{0}}\right)\times 100, \end{array}$$

where *A*_*t*_ is the absorbance at *t* = 5 and *A*_0_ is the absorbance at *t* = 0.

*(2) Coaggregation Test*. The coaggregation ability for each LAB isolate was analyzed using five pathogenic bacteria, namely, *Staphylococcus aureus* ATCC 6537, *Salmonella* sp. ATCC 14028, *Escherichia coli* ATCC 25922, *Shigella* sp. 31056, and *Staphylococcus epidermidis* ATCC 12228 which were grown in MRS broth and TSB media at 37°C for 18 hours. The suspensions were centrifuge at 3500 rpm for 20 minutes, after which each precipitate was washed twice and suspended into PBS until it reached 10^8^ CFU/mL. Two mL of each suspension was transferred to a sterile tube, while a total of 4 *μ*L of each bacterial isolate was placed into a sterile tube as a control. *Coaggregation* was measured at the initial and final absorbance (0 and 5 hours) after incubation at room temperature. Each bacteria suspension (0.1 mL) was added to 3.9 mL PBS and measured by a *spectrophotometer* (TECAN Infinite 200 PRO NanoQuant) at a wavelength of 600 nm. The percentage of *coaggregation* is stated as follows (Angelov et al., 2015):

(2) $$\begin{array}{c} Coaggregation\left(\%\right)=\frac{\left[\left(\left(\left(A_{x}+A_{y}\right)/2\right)–A_{x+y}\right)\right]}{\left(A_{x}+A_{y}\right)\times 100}, \end{array}$$

where *A*_*x*_ is the suspension absorbance of LAB isolates; *A*_*y*_ is the suspension absorbance of pathogenic isolates; *A*_*x*+*y*_ is the absorbance of the mixed suspension of LAB isolates and pathogens.

### 2.4. Identification of Selected Isolates from Wadi Papuyu

#### 2.4.1. Identification of Selected Isolates Using VITEK 2 Compact

Identification of selected isolates was performed on the isolate suspension using a VITEK 2 Compact system (bioMerieux®).

#### 2.4.2. Identification of Selected Isolates Using 16S and 18S rRNA-Gene Sequencing

Identification was performed using 16S rRNA and 18S rRNA-sequencing at Macrogen, Singapore. PCR primers used for amplification of bacterial 16S rRNA gene were 27F (5′-AGA GTT TGA TCM TGG CTC AG-3′); 1492R (5′-TAC GGY TAC CTT GTT ACG ACT T-3′), while the following PCR primers used for amplification of yeast gene were NS1 (5′ -GTA GTC ATA TGC TTG TCT C-3′); NS8 (5′-TCC GCA CGT TCA CCT ACG GA-3′). Sequencing primers used for bacteria were 785F 5′(CGA TTA GAT ACC CTG GTA)3′; 907R 5′ (CCG TCA ATT CMT TTR AGT TT)3′, while the following sequencing primers used for yeast were NS1 5′ (GTA GTC ATA TGC TTG TCT C)3′; N58 5′ (TCC GCA GGT TCA CCT ACG GA)3′.

## 3. Result

### 3.1. Preparation of Wadi

Wadi papuyu is made from fresh papuyu fish (*Anabas testudineus* Bloch.). The wadi papuyu fermentation process was carried out for 8 days under aerobic conditions in a tightly closed container. On the 8th day, the container was opened, and an organoleptic examination was carried out; the results obtained were fermented papuyu fish which were semi-wet, brown to black, and had the distinctive odor of fermentation (sour) (Figure 1).

### 3.2. Isolation of Bacteria during Fermentation of Wadi

Wadi papuyu isolates were grown on two media, MRS agar and MHA media. The number of microbial isolates that grew on MRS agar was 12 isolates and 10 isolates on MHA media. A total of 22 microbial isolates were then purified based on gram staining and morphology of the isolates. The purification results obtained 4 microbial isolates with codes MRS AKP, MRS AKK, MHA KP, and MHA KK. After purification, the microbial isolates were stored at -20°C in 15% glycerol and 85% medium (MRS broth and MH broth).

### 3.3. Characterization of Bacterial Isolates from Wadi

Further morphological and biochemical tests for isolates including gram staining, catalase test, viability test of bacterial isolates at *acid tolerance*, and *aggregation tests* (*autoaggregation* and *coaggregation*) were performed.

The results of morphological and biochemical characterization of isolates from wadi papuyu are summarized in Table 1.

### 3.4. Testing of Bacterial Isolates as Probiotics by In Vitro

#### 3.4.1. Acid Tolerance

Observations were made on the increase in absorbance of microbial isolates at pH 2, 3, and 4 and the number of isolates growing on MRS agar and MHA (Table 2).

#### 3.4.2. Aggregation Tests

*(1) Autoaggregation Test*. In the *autoaggregation test*, no significant increase was observed in the absorbance of the four bacterial isolates. Furthermore, the isolates MHA KK and MRS KP showed decrease in absorbance after 24 hours of incubation (Table 3).

*(2) Coaggregation Test*. For the *coaggregation* test, MHA KK isolates showed the best results against all tested pathogenic bacteria, especially *Staphylococcus epidermidis* with a percentage of 91.8%. Isolates MHA KP, MRS AKP, and MRS AKK yielded percentages ranging from 35% to 76%. Therefore, all tested bacterial isolates can compete with pathogenic bacteria in the gastrointestinal tract (*coaggregation*) (Table 4).

### 3.5. Identification Selected Isolates from Wadi Papuyu

#### 3.5.1. Results of the Identification Using VITEK 2 Compact

There are 4 cards used for microbial identification, namely, ANC (*Anaerobic* and C*orynebacterium*) card to identify *Lactobacillus garvieae*, GP (Gram-positive) card to identify *Staphylococcus warneri*, BCL card to identify *Bacillus pumilus*, and YST card to identify *Candida lusitaniae.*

#### 3.5.2. Results of Identification Using 16S rRNA and 18S rRNA-Gene Sequencing

**Figure 2:**
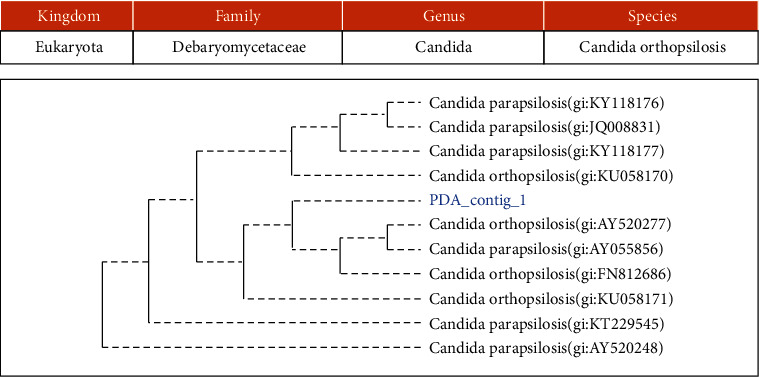
The phylogenetic study of isolate PDA/MRS AKP.

**Figure 3:**
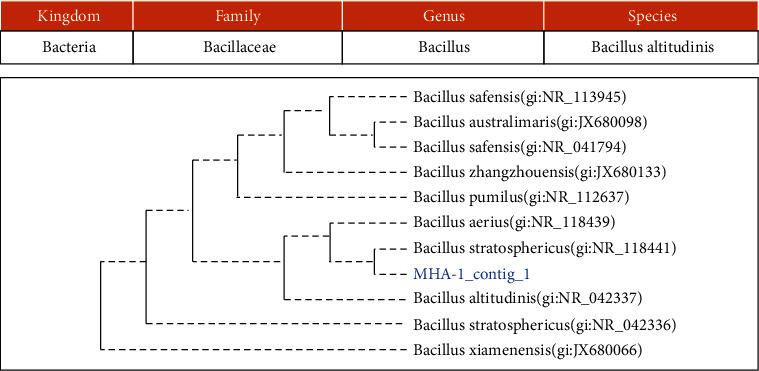
The phylogenetic study of isolate MHA-1/MHA KK.

**Figure 4:**
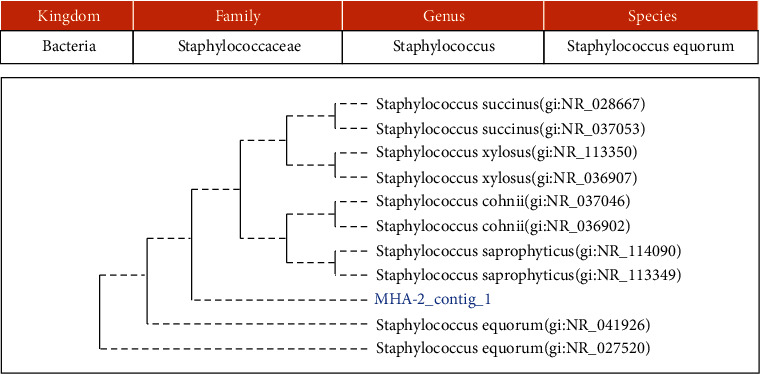
The phylogenetic study of isolate MHA-2/MHA KP.

**Figure 5:**
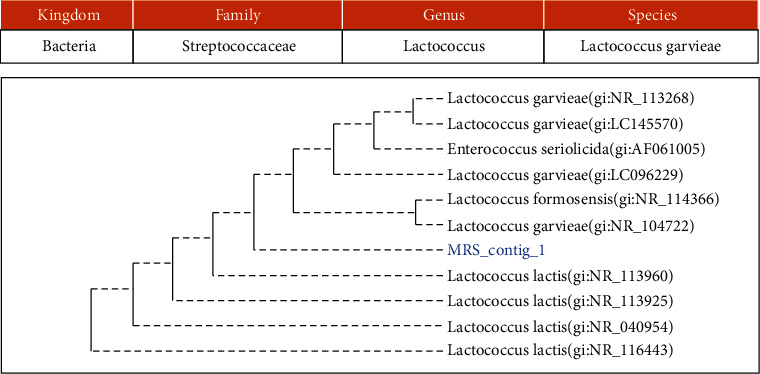
The phylogenetic study of isolate MRS-1/MRS AKP.

Using 16S rRNA and 18S rRNA-gene sequencing, 3 bacteria and 1 yeast were identified, namely, *Lactococcus garvieae*, *Bacillus altitudinis*, *Staphylococcus equorum*, and *Candida orthopsilosis* (Figures 2, 3, 4, and 5).

## 4. Discussion

Wadi of papuyu is produced using fresh fish (without any drying process) with the internal organs removed. In this process, the salting method is used, where the salt is about 25% of the total weight of the processed fish. This is in line with the statement of Khairina et al. (2006) that wadi is processed with the addition of 25% salt at room temperature for a minimum of 7 day. Wadi is created by adding *samu/lumu* (roasted-mashed rice). *Samu/lumu* contains starch as a source of carbohydrates. Fermented food products will utilize lactic acid bacteria in the process of making these products. Carbohydrates derived from starch will be utilized by LAB as an energy source [25, 26]. Bacterial isolation from wadi papuyu was performed using two different media, MRS and MHA. MRS media is widely used for the growth of LAB and the isolation of fermentation products [27–32]. MHA media was used because it is the most appropriate for sensibility tests (by the Kirby-Bauer method) on nonfastidious bacteria (both aerobic and facultative anaerobic bacteria). It is not a selective or differential medium and can grow all bacteria. According to Al-Shaffar and Jarallah [33], to isolate *Pseudomonas aeruginosa* from a hospital environment in the province of Babylon, several growth media were used, such as blood agar base, MacConkey agar, MHA, and nutrient broth.

Negative results were obtained from the catalase test for the MRS AKP isolate; this was similar to LAB isolates of probiotic candidates from the digestive tract of broiler chickens [34]. Tolerance to acidic test of Labi cattle gastric LAB isolates showed resistance to low pH once OD (optical density) ≥ 0.1 and no resistance at low pH once OD ≤ 0.1 [35]. One of the requirements for bacterial isolates to be probiotic is to be resistant to various pH conditions, especially low pH (e.g., the pH of gastric acid). Also, LAB as probiotics will experience slow growth at low pH, because bacterial cells can get damaged under acidic conditions and lose their viability. Therefore, each bacterial isolate has a different resistance to high and low pH. Tolerance to acidic conditions is an important characteristic for bacterial isolates passing through the gastrointestinal tract [36]. Acidic conditions in the digestive tract are a natural barrier to most microorganisms from entering the intestine. The ability to survive and grow in a low pH environment is a characteristic of LAB and fungi or yeasts [37].The autoaggregation test aims to measure the ability of bacteria to adhere and multiply in the digestive tract. Moreover, this is an important step in the selection of probiotic strains, as it is a prerequisite for colonization of the gastrointestinal tract [38]. The coaggregation test aims to evaluate the ability of LAB isolates to inhibit pathogenic bacteria in the digestive tract. One of the effects of probiotics is the “barrier” effect. Barrier effect is called competitive exclusion [39]. This effect provides resistance against pathogenic bacteria by preventing or inhibiting colonization by these bacteria (Angelov, 2015). The coaggregation test is important to evaluate the effect of LAB on the defense mechanism of the body, especially the digestive tract against infection with pathogenic bacteria. Sufficient amounts of LAB, such as in *Lactobacillus* sp., are believed to be able to create a balance of healthy and beneficial microflora in the digestive tract [40].

The results of in vitro characterization were obtained using VITEK 2 Compact system. The VITEK 2 Compact also allows for rapid screening of microorganisms against antibiotic resistance which can improve clinical outcomes and reduce costs [41]. Using VITEK 2 Compact can save identification time as it only takes about 3 to 24 hours, compared to traditional methods which can take up to 48 hours [42]. Furthermore, 16S rRNA and 18S rRNA analysis are the methods of choice for the identification of new isolates especially to the species level with high sensitivity and specificity. This molecular-based identification method uses sequencing analysis and requires a shorter time than the conventional methods [24].

Identification which was carried out using the VITEK 2 Compact system and PCR-sequencing methods both produced different yeast species. Several yeasts were also obtained in other studies using the PCR-sequencing method, one of which was *Candida* sp. The *Candida* sp. group was found in fermented fish including *Candida parapsilosis*, *Candida orthopsilosis*, *Clavispora lusitaniae*, and *Rhodotorula mucilaginosa* [43].

## 5. Conclusion

A total of 3 bacterial isolates and 1 *yeast* were identified from wadi made of papuyu (*Anabas testudineus* Bloch.) with the VITEK 2 Compact system (*Lactococcus garvieae*, *Bacillus pumilus*, *Staphylococcus warneri*, and *Candida lusitaniae*.) and 16S rRNA and 18S rRNA-gene sequencing (*Lactococcus garvieae*, *Bacillus altitudinis*, *Staphylococcus equorum*, and *Candida orthopsilosis*). Furthermore, the *in vitro* tests results especially those from the *autoaggregation* test, showed that none of the bacterial isolates could be used as probiotic candidates.

## Figures and Tables

**Table 1:** Bacterial isolate characterization results from wadi of papuyu.

Bacterial isolate code	Characterization of bacterial isolates		
	Gram staining	Bacterial form	Catalase test
MHA KK	Purple (+)	Bacilli	+
MHA KP	Purple (+)	Cocci	+
MRS AKP	Purple (+)	Cocci	—
MRS AKK	Purple (+)	Cocci	+

**Table 2:** *Acid tolerance* results.

Bacterial isolate code	Microbial isolates growth			Average number of bacterial isolates on media	
	OD at *λ* 660 nm			Before incubation	After incubation
	pH 2	pH 3	pH 4	pH 2	
MHA KK	0.123	0.222	0.167	13	56
MHA KP	0.203	0.147	0.158	11	43
MRS AKP	0.174	0.14	0.127	18	96
MRS AKK	0.174	0.244	0.195	24	60

**Table 3:** *Autoaggregation test* results.

No.	Bacterial isolate code	A_0_	A_24_	*Auto-aggregation* percentage (%)	Conclusions
1	MHA KK	0.044	0.044	0	*Non-autoaggregation*
2	MHA KP	0.045	0.042	6,7	*Non-autoaggregation*
3	MRS AKP	0.044	0.044	0	*Non-autoaggregation*
4	MRS AKK	0.042	0.040	4.8	*Non-autoaggregation*

**Table 4:** *Coaggregation test* results.

No.	Bacterial isolate code	Pathogenic bacteria	*Coaggregation* percentage (%)
1	MHA KK	*Staphylococcus aureus*	64.0
2	MHA KP		37.1
3	MRS AKP		60.0
4	MRS AKK		37.3
5	MHA KK	*Escherichia coli*	58.5
6	MHA KP		41.3
7	MRS AKP		59.0
8	MRS AKK		35.0
9	MHA KK	*Staphylococcus epidermidis*	91.8
10	MHA KP		60.9
11	MRS AKP		76.2
12	MRS AKK		65.9
13	MHA KK	*Shigella* sp.	73.4
14	MHA KP		46
15	MRS AKP		75
16	MRS AKK		73.4
17	MHA KK	*Salmonella* sp.	71.2
18	MHA KP		47.5
19	MRS AKP		60.0
20	MRS AKK		50

## Data Availability

Data are available on request due to privacy or other restrictions.

## Additional Points

*Publisher's Note.* This journal remains neutral with regard to jurisdictional claims in published institutional affiliation.
